# Implementation and Outcomes of Multidisciplinary Diabetes Management Program Among Type 2 Diabetic Patients: A Comparative Study

**DOI:** 10.7759/cureus.60979

**Published:** 2024-05-24

**Authors:** Youssef H Ahmed, Hussain s Elbadawi, Intessar Sultan, Rehab A Mohammed, Huda Aljedaani, Hanaa E Abozeid, Mayar Badawy

**Affiliations:** 1 Department of Health Sciences, Syreon Middle East LLC, Alexandria, EGY; 2 Metabolic Unit, My Clinic International, Jeddah, SAU; 3 Department of Internal Medicine, Ibn Sina National College for Medical Studies, Jeddah, SAU; 4 Department of Internal Medicine, Faculty of Medicine for Girls, Al-Azhar University, Cairo, EGY; 5 Department of Obstetrics and Gynecology, Ibn Sina National College for Medical Studies, Jeddah, SAU; 6 Department of Clinical Pharmacy, Faculty of Pharmacy, Tanta University, Tanta, EGY

**Keywords:** personal health coordination, diabetes care, multidisciplinary health care, physician-led care, t2dm, type 2 diabetes

## Abstract

Background: Current guidelines recommend shifting physician-led care (PLC) for type 2 diabetes mellitus (T2DM) to more effective multidisciplinary health care (MHC). However, few researchers have studied its real-life implementation in Saudi Arabia. Therefore, we aimed to assess the implementation and compare the outcomes of an MDC diabetes management program (DMP) among T2DM patients to a PLC at a general hospital after one year of follow-up in a real-world practice setting.

Methods: We conducted this comparative patient files review study by analyzing medical records of all T2DM patients at two private care centers. Both were compared for their effectiveness in achieving two outcomes: the glycated hemoglobin (HbA1c) <7% and low-density lipoprotein-cholesterol (LDL-c) <70 mg/dl at the end of the first year. Additionally, we assessed the implementation of the DMP.

Results: Eight hundred thirty-four medical records were reviewed, 537 from DMP, and 279 from the PLC center. The personal health coordination was almost complete (97.8%) in the DMP, but the implementation was incomplete regarding nutrition (65.7%), dental exam (64.8%), and foot care (58.3%). Both care groups were matched for age (p = 0.056), gender (p = 0.085), duration of diabetes (p = 0.217), and basal glycemic control (p = 0.171). The DMP showed a significant net decrease in HbA1c (-0.5 [IQR 1.47%] vs -0.2 [IQR 3.05%], p = 0.0001) and LDL-c (-10 [IQR 50] vs -5 [IQR 60.5] mg/dl, p = 0.004) compared to PLC. A higher percentage of patients achieved glycemic control in the DMP than in the PLC (49.4% vs 38.7%, p = 0.038). However, both programs demonstrated similar outcomes in lipid control (28.7% vs. 30%, p = 0.695).

Conclusion: Despite some gaps in implementation, one year of DMP showed better glycemic control among T2DM patients compared to PLC. Both programs were comparable in terms of lipid control. Further studies identifying the gaps in care implementation could improve sustainability, future replication, and generalizability of similar programs to other healthcare systems in Saudi Arabia.

## Introduction

Diabetes management faces many challenges that include both patient-related factors [1] and provider-related clinical inertia. Clinical inertia is seen in the delay of intensifying management due to poor glycemic control [2]. The Chronic Care Model is a successful framework that promotes the quality of diabetes care [3]. One of its core elements is the delivery system design wherein scheduled visits are coordinated through expanding the team-based approach to intensify disease management strategies, especially for patients with poor metabolic control, incorporating care management teams comprising dietitians, nurses, pharmacists, and other healthcare professionals [4].

Various health authorities [5,6] recommend shifting the diabetes care approach from conventional physician-led care (PLC) to the multidisciplinary health care (MHC) model. While PLC is non-integrated care, the MHC engages patients with a professional health team for optimal diabetes management care through the implementation of a healthy lifestyle as well as proper therapeutic interventions [7]. The health team for type 2 diabetic patients should include a diversity of relevant specialties, such as diabetes nurse educators, psychologists, clinical pharmacists, nutritionists, podiatrists, and internists/endocrinologists.

Results from different countries reported a positive impact of MHC on the patient's adherence to medical and self-care [8,9]. Compared with PLC, MHC has been associated with improved glycemic control, blood pressure, lipid profile, and quality of life in type 2 diabetes mellitus (T2DM) patients [10,11]. Moreover, it has shown a significant reduction in diabetic foot complications [12], end-stage renal disease in patients with diabetic nephropathy [13], and risk of mortality [14].

Although the MHC approach has gained global acceptance for its effectiveness among T2DM patients, the most robust evidence comes from developed countries [7-14]. Saudi Arabia (SA) is among the world’s top 10 diabetes-prevalent countries [15], especially among young individuals [16]. Its growth rate is alarming, reaching up to 12%, and is expected to rise to 13.3% by 2030 [15]. This represents a major health hazard that necessitates the implementation of the best diabetes care to limit the diabetic social and economic burden in the kingdom. Studies addressing the impact of MHC on Saudi patients are missing. One Saudi study reported serial glycated hemoglobin (HbA1c) reduction among young diabetics under the impact of the MHC approach [17].

The major diabetes treatment goals are HbA1c and low-density lipoprotein cholesterol (LDL-C) levels. Therefore, our study was performed to compare the improvement of HbA1c and LDL-C among T2DM patients under the MHC diabetes management program (DMP) versus PLC in a clinical record review study.

## Materials and methods

Design

This was a record review comparative study conducted in two healthcare centers in Jeddah, KSA: an MHC and a standard PLC. Both centers belong to the private sector of the Saudi Ministry of Health.

Diabetes care

T2DM patients attending the "My Clinic" center received an MHC team approach through a prespecified "DMP" that offers personalized care. The personal health coordinator reminds patients of their lab tests, medical consultation dates, and follow-up on progress. It facilitates access to program services, including lab tests, weekly glucose log booklet, medication refill, lifestyle management, health education, dietician services and customized healthy-eating plan, eye checks, foot examinations, dental check-ups, and annual vaccination. Medical consultations are arranged at three-month intervals by an internist or endocrinologist. Dental, ophthalmic, and foot examinations, renal function, and albumin/creatinine ratio are arranged on a yearly basis. HbA1c is planned to be measured every four months, and a complete lipid profile (total cholesterol, triglycerides, high-density lipoprotein cholesterol, calculated LDL cholesterol) every six months. The healthcare team includes internists, endocrinologists, diabetes nurse educators, dietitians, clinical pharmacists, and medical social workers for counseling.

The PLC group received standard care at a general college hospital. This care group was monitored at variable intervals by internists or endocrinologists. Each clinic visit independently assesses the needs of the patients and selects the time for the needed follow-up investigations and other consultations according to the local hospital policy and procedure. All doctors had unrestricted access to laboratory tests. There was no definite protocol to ensure the compliance of patients and medical staff.

Process of clinical record review

First: Identifying an Appropriate Data Source

The identification of clinical case records in the outpatient service. All electronic records of T2DM patients who completed one year of follow-up from June 2022 to June 2023.

Second: Devising a Data Extraction Instrument

An Excel sheet was designed by experienced information technologists at both centers to extract data from medical records. After the construction of Excel sheets, researchers excluded patients under basal-bolus insulin therapy as glycemic control in these patients is best evaluated by self-monitoring of blood glucose or continuous glucose monitoring, in addition to HbA1c. Patients with diseases associated with HbA1c variability (hemolytic and other anemias, glucose-6-phosphate dehydrogenase deficiency, use of drugs that stimulate erythropoiesis, end-stage renal disease, and pregnancy) were also excluded.

Third: Extraction of the Data

Three researchers carried out data extraction in accordance with the agreed-upon definitions. Other researchers reassessed a dataset sample to check agreement with the previous data and to determine the pattern and extent of inaccuracies, if any. Variables included age, gender, components of diabetes care, and laboratory results of two metabolic parameters (HbA1c and LDL-C). The goal is to achieve HbA1c <7% and LDL-C <70 mg/dl at the end of the first year [18]. 

Statistical analysis

The data were analyzed using the Statistical Package for the Social Sciences (IBM Corp. Released 2013, IBM SPSS Statistics for Windows, version 22.0. Armonk, NY). Categorical variables were represented as percentages and frequencies, while numerical variables were summarized by calculating the median and interquartile range due to their abnormal distribution. Non-parametric-related samples Friedman test was used to compare the four levels of HbA1c and LDL-C. Wilcoxon signed ranks test was used to compare the difference in HbA1c and LDL-C levels at the end of year 1 with Z based on positive ranks. Chi-square and non-parametric Mann-Whitney U tests were used to compare different groups. Multivariate linear regression was used to determine the significant models for prediction. All results were considered statistically significant at a p-value <0.05.

The minimal sample size of 100 patients required for this study was computed using G*power 3.1.9.4, given alpha of 0.05, power 95%, effect size of 0.25, and linear multiple regression as the statistical test with the number of predictors of 5.

## Results

A total of 834 medical records were reviewed in the study (DMP, n = 537; PLC, n = 297). Table 1 shows the implementation of the DMP after the completion of the first year with almost complete personal health coordination (97.8%). The limitation of implementation of the program was observed mainly in medical nutrition therapy (65.7%), dental exams (64.8%), and foot care (58.3%).

**Table 1:** Implementation of the diabetes management program (DMP) as a multidisciplinary diabetes care after completing the first year at "My Clinic" center

				DMP completed year 1, N = 537	
				N	%
Personal health coordination	No		12		2.2%
	Done		525		97.8%
Completed all visits/Consultations	No		108		20.1%
	Done		429		79.9%
Ophthalmic exam	No	232			43.2%
	Done	305			56.8%
Foot care	No	313			58.3%
	Done	224			41.7%
Dental exam	No	348			64.8%
	Done	189			35.2%
Medical nutrition therapy	No	353			65.7%
	Done	184			34.3%

Table 2 compares the efficacy of the two programs on HbA1c and LDL-C at the end of the first year. Both care groups were matched for the patients' median age of 49 (12) vs. 50 (11) years (p = 0.056), gender (males 71.4% vs. 65.5%, p = 0.085), and duration of diabetes 8 (11) vs. 8 (10) years, p = 0.217. The HbA1c changes were insignificant in PLC (p = 0.158) after a one-year follow-up, but it was significant in the DMP (p < 0.029). The DMP showed a significant net decrease in HbA1c (-0.5 [IQR 1.47%] vs. -0.2 [IQR 3.05]%, p = 0.0001). However, both groups had significant LDL-C changes (p = 0.029 and p < 0.001). The DMP showed a significant net decrease in LDL-C (-10 [IQR 50] mg/dl) compared to PLC (-5 [IQR 60.5], p = 0.004).

**Table 2:** Comparison between the care approaches among T2DM patients who completed one year of diabetes care IQR: interquartile range; T2DM: type 2 diabetes mellitus; LDL: low-density lipoprotein.

		Type 2 diabetes care N = 834		p-Value
		Physician-led care N = 297	Diabetes management program N = 537	
Gender	Female	85 (28.6%)	185 (34.5%)	0.085
	Male	212 (71.4%)	352 (65.5%)	
Age: median (IQR): years; Min-Max		49 (12); 32-87	50 (11); 33-80	0.056
Duration of diabetes: median (IQR): years; Min-Max		8 (11); 3-30	8 (10); 3-30	0.217
Basal A1C: median (IQR) %		7.7 (2.9)	7.3 (2.3)	0.006
Follow-up A1C: median (IQR) %		7.6 (2.6)	6.9 (1.6)	<0.001
Median change (IQR) %; Significance of the change: p-value		0.2 (3.05); p = 0.158	0.5 (1.47); p < 0.001	0.001
Basal LDL-cholesterol: mg/dl		100.5 (51)	107 (59.5)	0.020
Follow-up LDL-cholesterol: mg/dl		91.5 (44.25)	95 (50.5)	0.300
Change: median (IQR): mg/dl; Significance of the change: p-value		5 (60.5); p = 0.029	10 (50); p < 0.001	0.004

Table 3 shows the metabolic control at basal and after one-year follow-up of two different diabetes care centers. While basal glycemic levels were matched between the two approaches, after one year of care, DMP showed a significantly higher percentage of controlled patients (49.5% vs 38.7%, p < 0.001). Despite basal lipid control being better among the PLC (p = 0.001), both approaches showed comparable outcomes after one year of care (p = 0.695).

**Table 3:** Comparison of the glycemic and LDL-C control at basal and after one year of two different diabetes care approaches LDL-C: low-density lipoprotein-cholesterol.

			Physician-led care (n = 297)	Diabetes management program (n = 537)	p-Value
Glycemic control	Basal	Controlled (<7%)	104 (35.0%)	183 (34.1%)	0.171
		Uncontrolled (7-8%)	65 (21.9%)	148 (27.6%)	
		Poorly uncontrolled (>8%)	128 (43.1%)	206 (38.4%)	
	After 1 year of follow-up	Controlled (<7%)	115 (38.7%)	266 (49.5%)	<0.001
		Uncontrolled (7-8%)	56 (18.9%)	161 (30.3%)	
		Poorly uncontrolled (>8%)	126 (42.4%)	110 (20.5%)	
	Comparison between basal and follow-up		p < 0.001	p < 0.001	
LDL-C control	Basal	Controlled (<70 mg/dl)	81 (27.3%)	94 (17.5%)	0.001
		Uncontrolled (≥70 mg/dl	216 (72.7%)	443 (82.5%)	
	After 1 year of care	Controlled (<70 mg/dl)	89 (30%)	154 (28.7%)	0.695
		Uncontrolled (≥70 mg/dl	208 (70%)	383 (71.3%)	
	Comparison between basal and follow-up		p = 0.179	p < 0.001	

**Figure 1:**
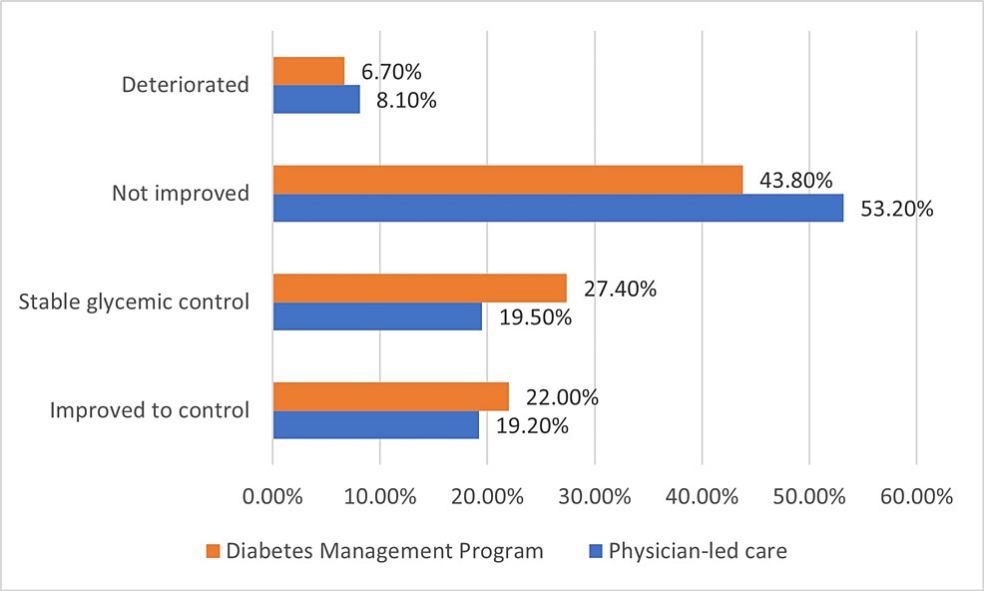
Glycemic control (HbA1c < 7%) after one year of follow-up of diabetes management program compared to physician-led care (not improved) (p = 0.024).

**Figure 2:**
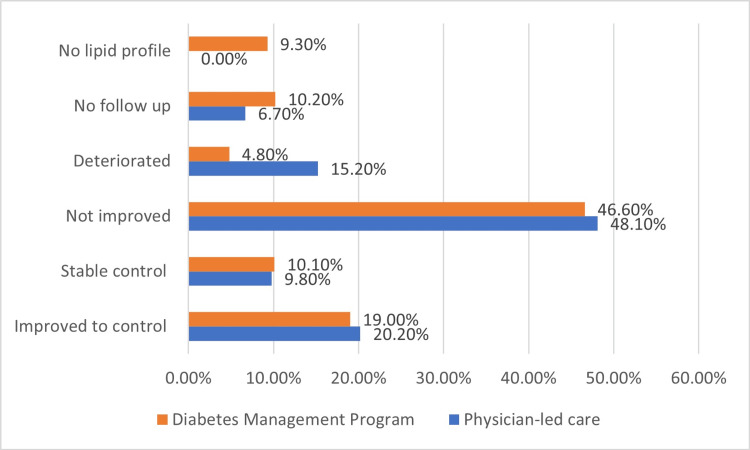
Low-density lipoprotein cholesterol control (<70 mg/dl) after one year of follow-up of diabetes management program compared to physician-led care (p < 0.001).

Details of changes are seen in Figures 1, 2. The glycemic control at the end of year 1 of DMP was not improved in 43.8% or even deteriorated in 6.7% of patients. Only 22% improved to control, and 27.4% were controlled from the start and continued to be controlled; the total percentage of controlled patients was significantly higher compared to PLC (49.4% vs. 38.7%, p = 0.038) (Figure 1). The LDL-C control at the end of year 1 of DMP was not improved at 46.6%, improved at 19.0%, and stable from the start at 10.1% (Figure 1).

The total control rate (stable and improved) in the DMP was nearly equal to PLC (29.1% vs. 30%) with a lower percentage of patients who deteriorated (4.8% vs. 15.2%) (p < 0.001) (Figure 2).

In both care groups, age, gender, diabetes duration, type of care approach, and basal glycemic control significantly predicted glycemic control at the end of the first year (X^2^ = 135, p < 0.001, R^2 ^= 20.1%). This model could explain 20% of the variation of the glycemic outcome and correctly classify 69.2% of the controlled cases. The basal glycemic control (OR 5.50, 95% CI 4.00-7.57, p < 0.001) and receiving care at DMP (OR 1.68, 95% CI 1.22-2.30, p = 0.001) significantly predicted the achievability of glycemic control. Similarly, age, gender, duration, place of care, and basal LDL-C control significantly predicted the LDL-C control target at the end of the first year (X^2^ = 30.70, p < 0.001, R^2 ^= 5.2%). However, the model could explain only 5.2% of the variation of the LDL-C outcome and correctly classify 70.4% of the controlled cases. The basal LDL-C control (OR 5.50, 95% CI 4.00-7.57, p < 0.001) significantly predicted the achievability of the control (Table 4).

**Table 4:** Predictors of glycemic and lipid control at the end of one year of follow-up for both diabetes care approaches MHC: multidisciplinary health care; DMP: diabetes management program; LDL-C: low-density lipoprotein cholesterol.

		Exp (B) Odds ratio	95% Confidence interval		p-Value
			Lower	Upper	
Glycemic control	Gender (females/males)	0.856	0.85	1.61	0.337
	Age	0.989	0.97	1.01	0.372
	DM duration	1.029	1.00	1.06	0.077
	Basal glycemic control (controlled/uncontrolled)	5.50	4.00	7.57	<0.001
	Diabetes care (DMP/PLC)	1.68	1.22	2.30	0.001
LDL-C control	Gender	0.998	0.72	1.38	0.993
	Age	0.992	0.97	1.02	0.563
	DM duration	1.016	0.98	1.05	0.350
	Basal LDL-C control (controlled/uncontrolled)	2.65	1.87	3.76	<0.001
	Diabetes care (DMP/PLC)	1.04	0.76	1.44	0.796

Table 5 shows that in patients under DMP, age, gender, diabetes duration, basal glycemic levels, and attending regular ophthalmic visits significantly predicted the targeted glycemic outcome at the end of the first year (X^2^ = 122, p < 0.001, R^2 ^= 27.1%). Their model could explain 27% of the variation of the glycemic outcome and correctly classify 71.5% of the controlled cases. The basal glycemic control (OR 8.38, 95% CI 5.43-12.94, p < 0.001) and receiving ophthalmic care (OR 1.63, 95% CI 1.11-2.40, p = 0.001) significantly predicted the achievability of glycemic control. Therefore, basal glycemic levels and ophthalmic visits were the main significant predictors of glycemic outcome at the end of the follow-up period.

**Table 5:** Predictors of glycemic control at the end of year 1 of the diabetes management program

	Exp (B) Odds ratio	95% Confidence interval		p-Value
		Lower	Upper	
DM duration	1.03	0.99	1.07	0.164
Gender	0.91	0.61	1.37	0.659
Age	0.99	0.96	1.02	0.355
Basal glycemic control	8.38	5.43	12.94	<0.001
Ophthalmic examination	1.63	1.11	2.40	0.014
Consultations	0.74	0.46	1.20	0.218

## Discussion

In this study, DMP provided personalized health coordination to T2DM patients with some limitations during its implementation for one year. Based on the level of basal glycemic control, half of the T2DM patients were well-controlled. On the other hand, less than their third reached target LDL-C. Compared to PLC, the DMP demonstrated a better efficacy with a 0.5% net decrease of HbA1c and 10 mg/dl of LDL-C compared to 0.20% and 5 mg/dl in the PLC patients. Furthermore, the care provided by DMP predicted glycemic control but did not show the same effect on lipid control. 

The positive results of this real-world study replicated the beneficial impacts of interventions in controlled trials by employing the multidisciplinary team approach [19-22]. In this study, the magnitude of improvement in HbA1c of 0.5 in the DMP patients was higher than another similar study [19], which reported a net decrease of 0.2% after one year of follow-up despite having a comparable average basal HbA1c (7.2%) to our study (7.3%) reflecting the efficacy of our DMP. In another interventional study conducted in SA [20], a more significant reduction of 1.9% in HbA1c was reported. However, they included poorly controlled type 2 patients, and their evaluation focused mainly on the program’s short-term effects.

In our study, 28% of poorly controlled patients responded positively to the program, leading to a decrease in the percentage of poorly controlled patients (38.4% to 20.5%) compared to only 1.3% in the PLC group. The response of uncontrolled patients was augmented under MHC intervention, reaching up to 70% in another study [21,22]. Physicians tend to intensify their care when poorly controlled patients are encountered, which may augment the magnitude of improvement in glycemic control.

Taïeb et al. (2022) evaluated the effectiveness of an interdisciplinary approach including physical educators and dieticians in managing poorly controlled diabetes in 67 patients over one year, focusing on glycemic control and body weight. More than 93% of the participants responded positively to the interdisciplinary educational and therapeutic approach, with an average reduction in HbA1c of 1.73%. However, there was no significant decrease in BMI [23]. This may be attributed to the implementation gap regarding the dietician's consultation. This gap is also notable in our study as 65.7% of our patients didn't seek dietitian consultation reflecting poor adherence to dietary advice. 

The key finding demonstrating the program's clinical effectiveness was that almost half of the patients reached the treatment target as 22% improved and 27.4% maintained control after one year of care. For PLC, the proportion of patients who reached the control target was much lower (38.7%) despite the fact that their basal glycemic control was comparable.

The DMP involves various components of multidisciplinary care, including different visits of healthcare workers. In this study, apart from all personalized visits in the DMP group, ophthalmic consultation was the only predictor of the improved glycemic outcome, and it was also the highest frequency of visits, accounting for 56.8% among all other visits. These results could reflect the importance of adhering to all components of the diabetes care program to achieve positive results. 

Participants demonstrated a 10 mg/dl net reduction of LDL-C from the basal level. However, compared to PLC, there was no significant difference in the effectiveness of both programs regarding LDL-C. Notably, the baseline LDL-C levels were not equivalent between the DMP and PLC groups, with a significantly lower proportion of patients achieving their target in the DMP group. This disparity in baseline characteristics could mask the program's true impact on LDL-C and, therefore, explain the absence of significant differences between both groups after one year. These results may highlight the challenges in managing hyperlipidemia in some patients. Guidelines urge primary care providers to refer difficult hyperlipidemic cases to more specialized care with a multidisciplinary team. This team-based care has increased the likelihood of patients achieving target lipid goals [24,25].

Limitations

There are some limitations of this study. First, the DMP had no clearly defined educational component and lacked the specification of definite interdisciplinary roles of participating healthcare professionals. Second, the study lacked details on medications and treatment plans, which might have affected the outcomes. Third, the one-year follow-up duration was insufficient to validate the program's efficacy. The last limitation was the reliance on HbA1c as the sole test for judging glycemic control. Despite its variability in the measurement, HbA1c is the gold standard for glycemic control. HbA1c does not address short-term glycemic variability, daily blood sugar fluctuations, or hypoglycemic events, which are important for optimizing care [26].

## Conclusions

This comparative study revealed that a multidisciplinary DMP for patients with T2DM was effectively implemented, albeit with some implementation gaps noted. Over a one-year follow-up period, the program led to significant reductions in HbA1c levels and improvements in glycemic control. However, the lipid control showed less improvement, suggesting the potential need for a longer follow-up duration. These promising findings endorse the effectiveness of the DMP approach in managing patients with T2DM and underscore the importance of transitioning from PLC to multidisciplinary care in SA. Further investigations on the long-term effects of DMP are strongly encouraged. Addressing implementation gaps can enhance multidisciplinary program stainability and applicability across other healthcare systems in SA.
